# Complete Endogenous Retrovirus Genome Sequence from a Brazilian Vampire Bat (Desmodus rotundus)

**DOI:** 10.1128/MRA.01497-18

**Published:** 2019-01-10

**Authors:** Luciano Chaves Franco Filho, Rafael Ribeiro Barata, Jedson Ferreira Cardoso, Janaina Mota de Vasconcelos Massafra, Poliana da Silva Lemos, Lívia Medeiros Neves Casseb, Ana Cecilia Ribeiro Cruz, Marcio Roberto Teixeira Nunes

**Affiliations:** a Centro de Inovações Tecnológica, Instituto Evandro Chagas, Ananindeua, Pará, Brazil; b Seção de Arbovirologia e Febres Hemorrágicas, Instituto Evandro Chagas, Ananindeua, Pará, Brazil; c Instituto de Ciências Biológicas, Universidade Federal do Pará (UFPA), Belém, Pará, Brazil; University of Rochester School of Medicine and Dentistry

## Abstract

The strain Desmodus rotundus *endogenous retrovirus* (DrERV) QR09 was obtained from a bat tissue sample collected from Desmodus rotundus in the Brazilian rain forest. The complete genome was sequenced using the next-generation sequencing strategy. The full-length genome of DrERV QR09 is 8,256 nucleotides in length and showed high similarity with other DrERVs.

## Abstract (precis)

The strain Desmodus rotundus *endogenous retrovirus* (DrERV) QR09 was obtained from a bat tissue sample collected from Desmodus rotundus in the Brazilian rain forest. The complete genome was sequenced using the next-generation sequencing strategy.

## ANNOUNCEMENT

Endogenous retroviruses (ERVs) are found in a wide variety of hosts and are possibly one of the first circulating viral strains of their respective hosts (1–3). ERVs that have integrated during host speciation, considered genetic fossils, are often used as markers for understanding the long-term phylogeny of the virus (4). In addition, several studies have shown that even when ERVs are inserted into the genome and considered inactive, there are indications that some proteins continue to be translated, even in low copies, and probably play important roles in cancer development and other gene regulatory activities (5–9).

The strain QR09 of Desmodus rotundus *endogenous retrovirus* (DrERV) was sequenced from a brain tissue sample of a bat (Desmodus rotundus) collected in the Brazilian Amazon rainforest (Viseu, Pará; 01°11′48″S, 46°08′24″W). The project was approved by the Ethics Committee on the Use of Animals of Evandro Chagas Institute (CEUA/IEC; number 031/2014) and Biodiversity Information and Authorization System (SISBIO; number 47592-1).

The viral particles were released from the cells using a stainless bead with a TissueLyser II (Qiagen), and the sample was preenriched using 0.45-µm filters and treatment with benzonase (25 U/liter). DNA and RNA were extracted with a iPrep PureLink virus kit (Thermo Fisher) following the manufacturer’s guidelines. The extracted DNA and RNA were quantified with a Qubit 2.0 fluorometer (Thermo Fisher) using the Qubit RNA HS assay kit, as well as the Qubit double-stranded DNA (dsDNA) HS assay kit (Thermo Fisher). The RNA samples were subjected to reverse transcription using the cDNA synthesis system kit (Roche, Branford, CT, USA), according to the manufacturer’s guidelines. The cDNA and DNA of brain tissue were combined and sequenced as a single sample. Sequencing libraries were constructed using the Illumina Nextera XT DNA sample preparation kit and sequenced on an Illumina HiSeq 2500 instrument with the high-output V4 2 × 100-bp sequencing kit (Table 1).

**TABLE 1:** DrERV strain QR09 sequencing information

Parameter	Strain information
Bat species	Desmodus rotundus
Tissue	Brain
Sequencing platform	Illumina
DNA/cDNA input (ng/μl)	1
Total no. of reads	241,897,814
Total no. of contigs	407,850
*N*_50_ (bp)	19,212

The raw data were filtered for Q30 quality, adapters were removed using Trim_galore pipeline v.0.4.5 (10), and reads less than 100 bp were removed using the Prinseqlite.pl tool (11). The removal of the rRNA sequences was performed using the SortMeRNA tool v.2.1b (12). The assembly was performed by IDBA-UD v.1.1.3 (13) using default settings. The comparison with the protein nonredundant protein database was carried out by the DIAMOND tool v.0.9.22.123 (14), with an E value of 0.00001 (15). The results were annotated using the Blast2GO tool (16).

The complete genome of DrERV strain QR09 is 8,256 nucleotides (nt) long, with 65-fold coverage and 48.6% GC content. Compared with the common structure of Betaretrovirus, the lineage shows the 4 common genes *gag* (2,187 nt), protease (837 nt), *pol* (2,418 nt), and *env* (1,770 nt). The *gag* and protease open reading frames did not present any stop codon; however, for the two coding regions *pol* and *env*, 2 and 3 stop codons were found, respectively. According to NCBI BLASTn analysis, the DrERV QR09 possesses 99% sequence identity over 100% query coverage of the DrERV isolate 824 (GenBank accession number KP175580) and 99% sequence identity over 96% query coverage of the DrERV isolate 216 (GenBank accession number KP175581); both genomes described were collected in Mexico from D. rotundus.

### Data availability.

The complete genome sequence of Desmodus rotundus *endogenous retrovirus* (DrERV) strain QR09 has been deposited in NCBI GenBank under the accession number MH648003. The sequencing reads (under Sequence Read Archive number SRR8208870) can be accessed through BioProject number PRJNA480298.
